# Disseminated cysticercosis with pulmonary and cardiac involvement

**DOI:** 10.4103/0971-3026.73532

**Published:** 2010-11

**Authors:** Bharat K Jain, Shilpa S Sankhe, Mukta D Agrawal, Prashant S Naphade

**Affiliations:** Department of MRI, KEM Hospital and Seth G. S. Medical College, Parel, Mumbai - 400 012, India

**Keywords:** Cardiac, disseminated cysticercosis, intraocular, pulmonary

## Abstract

Pulmonary and cardiac involvement by cysticercosis is extremely rare, and is usually asymptomatic. We report the case of a 19-year-old boy who presented with a history of headache and vomiting and was found to have disseminated cysticercosis with pulmonary and cardiac involvement; the emphasis is on the rare occurrence of pulmonary, cardiac, pancreatic, intraocular, and extradural spinal canal involvement in the same patient. This case demonstrates the extent to which cysticercosis can be disseminated.

## Introduction

Cysticercosis is an infection caused by the pork tapeworm *Taenia solium*. Infection occurs when the tapeworm larvae enter the body. Ingested eggs pass into the bloodstream and disseminate to various organs and form the cysts that characterize cysticercosis. Because cysticerci can be found anywhere in the body (most commonly in the brain and the skeletal muscles), their location and size determine the clinical presentation.[1]

Although disseminated cysticercosis has been described previously in a few case reports, there are no descriptions of the CT scan and MRI features of cysticercosis with cardiac, pulmonary, spinal, extradural, and ocular involvement to such an extent in the same patient.

## Case Report

**Figure 1:**
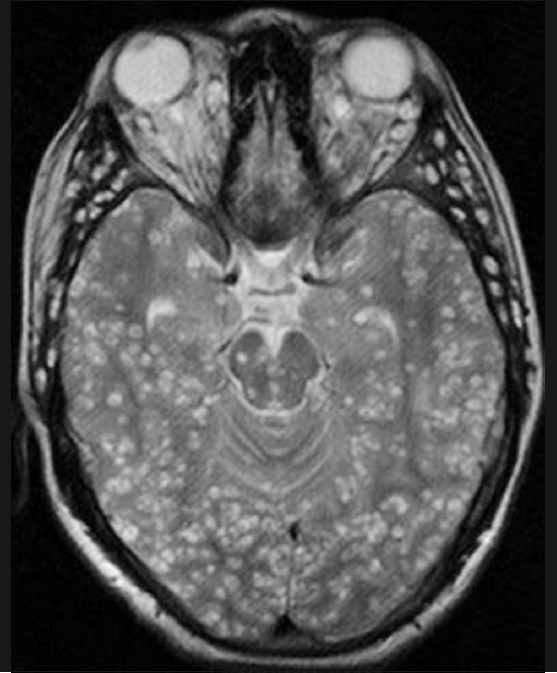
T2W axial MRI of the brain shows multiple cystic lesions with hypointense eccentric nodules, in both cerebral hemispheres, the midbrain, the cerebellum, and the extraocular muscles

**Figure 2:**
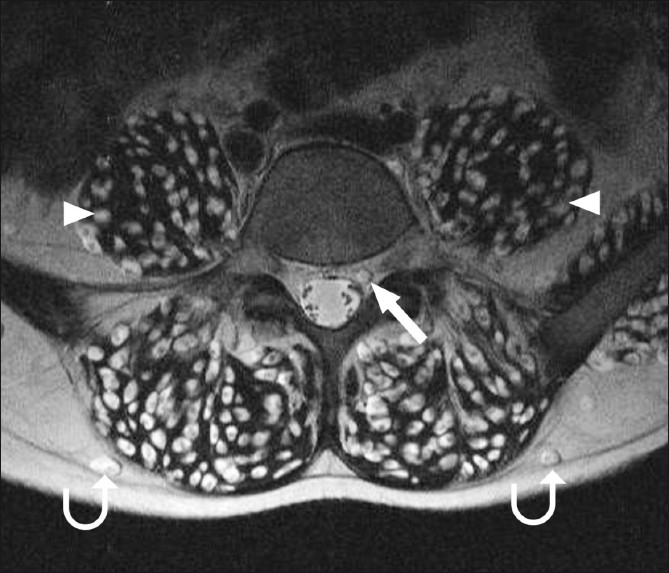
T2W axial MRI of the lumbar spine shows cysticercus cysts in the extradural spinal space (arrow), in addition to muscle (arrowhead) and subcutaneous (curved arrow) involvement

**Figure 3:**
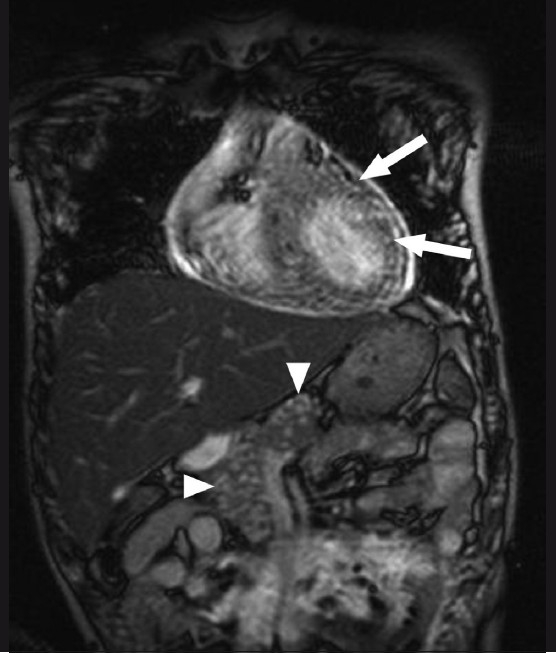
Balanced gradient MRI coronal image of the chest and upper abdomen shows hyperintense nodular lesions in the cardiac muscles (arrows) and pancreas (arrowhead)

**Figure 4:**
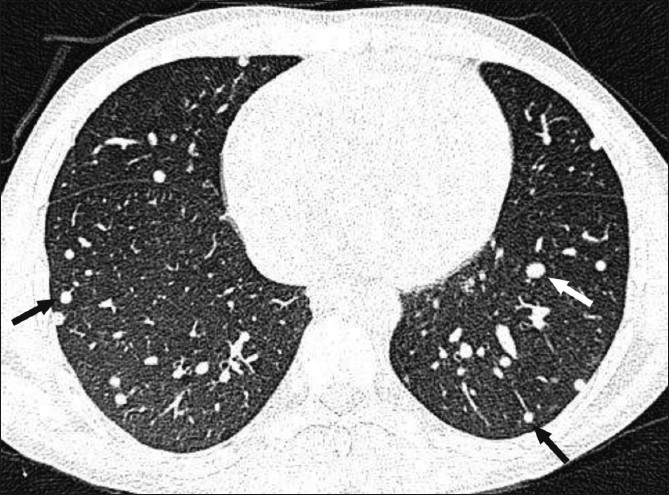
HRCT of the lungs shows multiple randomly distributed nodules (arrows) of varying sizes

**Figure 5:**
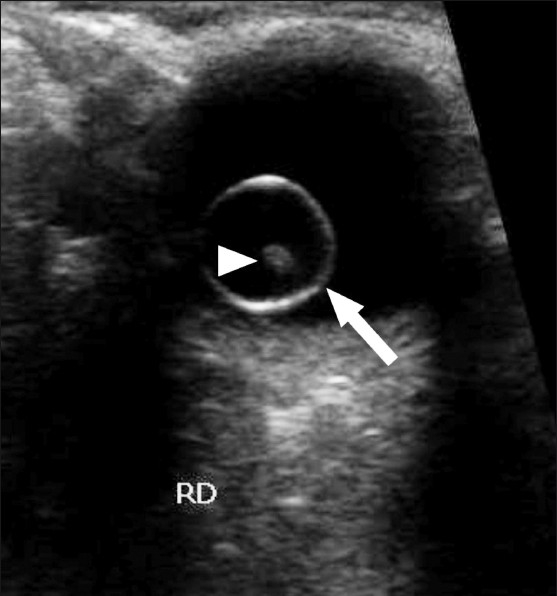
Transverse B-scan of the right eye shows a large intravitreal cyst (arrow) with a tiny hyperechoic scolex (arrowhead) within it

**Figure 6:**
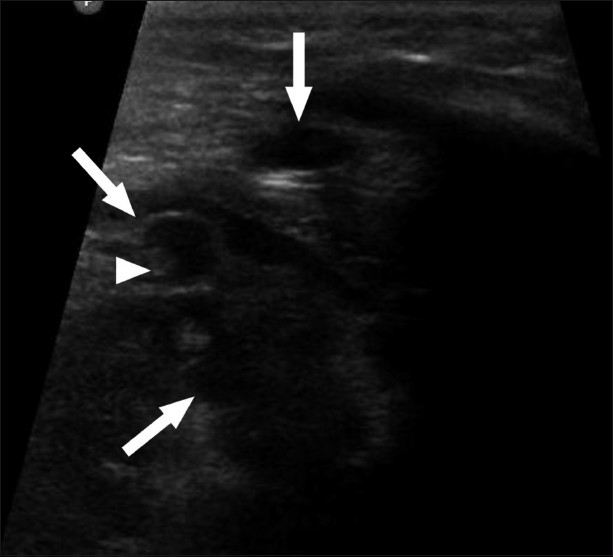
USG of the heart shows multiple cystic anechoic lesions (arrow) in the myocardium with a tiny hyperechoic scolex (arrowhead) within one of the cysts

A 19-year-old boy presented with a history of headache and vomiting for 6 months, seizures for 3 months, and decreased vision and bilateral proptosis (right more than left) for 1 month. The patient was apparently asymptomatic 6 months back. The patient was an Indian national and a vegetarian. Examination revealed subcutaneous nodules over the right eyelid, with mild proptosis of both eyes. Using the Snellen chart, his vision was found to be 6/30 in the right eye and 7/30 in the left eye. Neurological examination did not reveal any abnormality. All the laboratory investigations were normal. The patient was referred for MRI of the brain, which revealed multiple 3–8 mm-sized cystic lesions with T2-hypointense foci within both cerebral hemispheres, cerebellum, extraocular muscles of both orbits, and soft tissues of the neck [Figure 1]. Similar lesions were distributed in the muscles and adjacent subcutaneous tissues of the neck, back, abdominal wall, thighs, calves, legs, forearms and arms as well as the extradural spinal space [Figure 2]. Multiple cystic lesions were seen in both lungs and in the cardiac muscles [Figure 3]. Hyperintense nodules were also seen in the pancreas [Figure 3]. For further evaluation, a high-resolution CT (HRCT) of the lungs was performed, which revealed bilateral multiple, randomly distributed, 3-8 mm nodules [Figure 4]. A B-scan USG was performed, which revealed a large intravitreal cyst within the right eye, with a tiny hyperechoic scolex [Figure 5]. The left eye was normal. On superficial probe evaluation of the heart and pericardium through a parasternal window, using a high-frequency 7- to 12-MHz linear transducer (HDI 3000; Philips Medical Systems, Andover, MA, USA), there were multiple oval to circular, 0.5–1.5-cm sized, predominantly anechoic lesions in the heart muscle [Figure 6]. Later, a dedicated 2D echocardiography was performed, which revealed multiple disseminated cysticerci involving the muscle of the heart. On the basis of these imaging findings, a diagnosis of disseminated cysticercosis was made. The patient also had right bundle branch block on electrocardiogram; however, other functional parameters such as ejection fraction, valve function, systolic function and chamber size were normal on 2D echocardiography. A final diagnosis of cysticercosis was made and this was confirmed on muscle biopsy.

As drug-induced inflammation due to praziquantel may cause irreversible damage in cases with ocular and spinal lesions,[1] this drug was not given, and the patient was treated symptomatically with antiepileptic drugs, steroids, and diuretics.

## Discussion

Cysticercosis is an infection caused by the pork tapeworm, *Taenia solium*. The definitive host for T *solium* is man, who harbors the adult tapeworm; man as well as pigs act as intermediate hosts and harbor the larvae.[2,3] Infection occurs when the tapeworm larvae enter the body via contaminated food or water or through autoinfection. Ingestion of contaminated fruits, vegetables, and uncooked pork are known risk factors. The disease is also spread by contact with infected persons or fecal matter.[4] T. *solium* is present worldwide, but is most prevalent in Mexico, Africa, South-East Asia, Eastern Europe, and South America.[1]

The clinical symptoms of cysticercosis are protean, and basically reflect involvement of the affected organs. The most common occurrence of cysticerci is in the muscle, where it is usually asymptomatic. Intracerebral lesions frequently present with convulsions.[5] Decreased visual acuity or blindness can result from intraocular cysticerci. Cardiac lesions can cause arrhythmia. Paraparesis or gait abnormality may result from intraspinal lesions. Muscular pseudohypertrophy, a rare presentation, is caused by heavy infection of the skeletal muscles, which gives the patient a “Herculean appearance.” A few such cases have been reported, all from India.[6] Pulmonary and cardiac involvement in cysticercosis is rare. The diagnosis is usually confirmed by resolution of lesions following medical therapy with praziquantel or albendazole.[2] The radiological findings of cysticercosis - a cystic lesion with a central nodule that represents the scolex - are very similar in all affected organs. On MRI, cysticercosis lesions appear hyperintense, with well-defined edges, which show a hypointense eccentric nodule within, representing the dead parasite’s head and is called the scolex. The presence of a scolex in a cystic lesion usually suggests the diagnosis of cysticercosis.[7]

On HRCT, pulmonary lesions usually appear as multiple randomly distributed nodules of varying size. Radiologically, the lung lesions are nonspecific. The differential diagnosis includes disseminated tuberculosis, metastasis, and parasitic and fungal infections.[8] However, if involvement of other organs with cysticercosis is also seen, then this diagnosis can be entertained in the lungs as well. To visualize cranial and spinal involvement, MRI is the best modality.[9] To rule out intraocular involvement too, MRI is the modality of choice.[10] In our case, the patient presented with disseminated cysticercosis with cardiac, pulmonary, pancreatic, spinal, extradural, and ocular involvement. Not a single muscle of the body was spared. To the best of our knowledge, pulmonary and cardiac involvement has only rarely been described in the literature.[11] No study has described such an extent of involvement.

In conclusion, when a patient of cysticercosis presents with neurological symptoms, he/she should be evaluated for pulmonary and cardiac involvement in addition to all other body organs.
